# SNP-SNP Interaction in Genes Encoding PD-1/PD-L1 Axis as a Potential Risk Factor for Clear Cell Renal Cell Carcinoma

**DOI:** 10.3390/cancers12123521

**Published:** 2020-11-26

**Authors:** Marta Wagner, Krzysztof Tupikowski, Monika Jasek, Anna Tomkiewicz, Agata Witkowicz, Kuba Ptaszkowski, Pawel Karpinski, Romuald Zdrojowy, Agnieszka Halon, Lidia Karabon

**Affiliations:** 1 Laboratory of Genetics and Epigenetics of Human Diseases, Hirszfeld Institute of Immunology and Experimental Therapy, Polish Academy of Sciences, Ul. Weigla 12, 53-114 Wroclaw, Poland; marta.wagner@hirszfeld.pl (M.W.); monika.jasek@hirszfeld.pl (M.J.); anna.tomkiewicz@hirszfeld.pl (A.T.); ag.witkowicz@gmail.com (A.W.); 2 Subdivision of Urology, Wroclaw Comprehensive Cancer Center, Pl. Hirszfelda 12, 53-413 Wroclaw, Poland; tupikowski.krzysztof@dco.com.pl; 3 Department of Clinical Biomechanics and Physiotherapy in Motor System Disorders, Wroclaw Medical University, Ul. Grunwaldzka 2, 50-355 Wroclaw, Poland; kuba.ptaszkowski@umed.wroc.pl; 4 Department of Genetics, Wroclaw Medical University, 50-367 Wroclaw, Poland; polemiraza@poczta.fm; 5 Laboratory of Genomics and Bioinformatics, Hirszfeld Institute of Immunology and Experimental Therapy, Polish Academy of Sciences, 53-114 Wroclaw, Poland; 6 Department of Urology and Oncological Urology, Wroclaw Medical University, Ul. Borowska 213, 50-556 Wroclaw, Poland; romuald.zdrojowy@umed.wroc.pl; 7 Department of Pathomorphology and Oncological Cytology, Wroclaw Medical University, Ul. Borowska 213, 50-556 Wroclaw, Poland; agnieszka.halon@umed.wroc.pl; 8 Department of Pathomorphology, Wroclaw Comprehensive Cancer Center, Pl. Hirszfelda 12, 53-413 Wroclaw, Poland

**Keywords:** clear cell renal cell carcinoma, PD-1 (PDCD1), PD-L1, single nucleotide polymorphism

## Abstract

**Simple Summary:**

Immune checkpoints are key receptors that regulate the immune system and prevent its overactivation. This regulatory mechanism, which under normal conditions is responsible for maintaining immune homeostasis, can be misused by cancer cells, allowing them to avoid recognition and destruction. PD-1 is one of the major immune checkpoints that when interacting with its ligands—PD-L1/PD-L2, regulates the immune surveillance in the tumor microenvironment. We therefore hypothesized that single nucleotide polymorphisms (SNPs) (located in regulatory regions involved in regulation of expression and alternative splicing as well as SNPs introducing changes to the protein sequence) in genes encoding PD-1 and PD-L1 molecules may be associated with the development and outcome of renal cell carcinoma (RCC). We genotyped nine SNPs in PD-1/PD-L1 axis genes, with application of TaqMan allelic discrimination assays, and found that two of them taken together (rs10815225xrs7421861) may be considered to be potential risk factor for clear cell RCC.

**Abstract:**

PD-1/PD-L1 axis plays an important role in maintaining homeostasis and prevention from autoimmunity; however, in the tumor microenvironment, PD-1/PD-L1 interaction is responsible for the evasion of immune surveillance by tumor cells. We therefore hypothesized that single nucleotide polymorphisms (SNPs) in genes encoding PD-1 and PD-L1 molecules are associated with the development and outcome of renal cell carcinoma (RCC). Here we genotyped nine polymorphisms: five of *PDCD1*: rs36084323G>A, rs11568821G>A, rs2227981C>T, rs10204525G>A, rs7421861T>C and four of *PD-L1*: rs822335C>T, rs4143815G>C, rs4742098A>G, rs10815225G>C in 237 RCC patients (including 208 with clear cell RCC (ccRCC)) and 256 controls, with application of allelic discrimination method with use of TaqMan Assays. Interestingly, we found the SNP-SNP interaction between rs10815225 and rs7421861 polymorphisms associated with ccRCC risk. The rs7421861 TC genotype decreased the risk of ccRCC development compared to TT and CC genotypes in the group of rs10815225 GC + CC individuals (OR = 0.21, CI95% = 0.08; 0.54). While possessing of rs10815225 GC or CC genotype increased susceptibility to ccRCC when compared to rs10815225 GG genotype in individuals with rs7421861 TT or CC genotype (OR = 2.40, CI95% = 1.25; 4.61). In conclusion, genetic variants in *PDCD1* and *PD-L1* genes, especially taken together as SNP-SNP interactions, can be considered to be ccRCC risk factors.

## 1. Introduction

In 2000, Hanahan and Weinberg [1] established six hallmarks of cancer which include sustaining proliferative signaling, limitless replication potential, decreased sensitivity to antigrowth signals, evasion of apoptosis, activating invasion and metastasis, and promotion of angiogenesis. In 2011 the authors added two additional features which promote tumor development: reprogramming of energetic metabolism and second very important process—evasion from immune surveillance [2]. To avoid immune recognition cancer cells can use different mechanisms. One of them is development of an immunosuppressive tumor environment through expression of immune checkpoint molecules (ICs), such as CTLA-4, PD-1 and PD-L1, which negatively regulate immune response [3].

Programmed death receptor-1 (PD-1) is a member of CD28 family, expressed on double negative αβ and γδ T cells in thymus and activated T and B cells [4,5]. PD-1 molecule after interaction with its ligands—programmed cell death ligand-1 (PD-L1) and/or programmed cell death ligand-2 (PD-L2), limits some of the T cell functions, including T cell proliferation, apoptosis and IFN-γ production [6]. PD-L1 and PD-L2 are commonly expressed on endothelial cells, T and B cells, macrophages, dendritic cells, and stromal cells. Therefore, PD-1/PD-L1 interaction inhibits T cell function within tissue-specific sites in a late phase of immune response, what is crucial for the maintenance of long-term tolerance [5]. Importantly, PD-1 ligands can also be expressed by tumor cells and tumor stroma. The PD-1/PD-L1 interaction in the tumor microenvironment is directly responsible for the impaired immune response against cancer and for the evasion of immune surveillance by tumor cells. In this way PD-1/PD-L1 interaction may provide, mentioned above, immune escape mechanism for tumor cells [7]. To prevent this process, in recent years, highly efficacious immune checkpoint inhibitors (anti-CTLA-4 and anti-PD-1, alone or in combination), whose role is to enhance immune response against cancer cells, were introduced into clinical practice and they revolutionized cancer immunotherapy [8].

One of tumors which has long been categorized as “immunotherapy-responsive” is renal cell carcinoma (RCC) [9]. RCC is a common (the most frequent type of kidney cancer) and deadly disease—it was estimated that in 2018 there were approximately 400,000 new cases worldwide and ~175,000 deaths from RCC [10]. RCC comprises three major histological subtypes: clear cell RCC (ccRCC), papillary RCC and chromophobe RCC. The most common subtype is ccRCC (accounting for 70–80% of cases) which we have focused particularly on in our research [10].

Given the above mentioned literature data, it can be hypothesized that tumor development (including renal cell carcinoma) as well as the clinical course of the disease depend on the expression level of immune checkpoint molecules, inter alia members of PD-1/PD-L1 axis. It is well established that the mRNA and protein expression level can be regulated, among others, by the presence of genetic variants that can affect epigenetic modifications (methylation, microRNA binding), transcription factors binding sites and the formation of protein isoforms. Therefore, we put forward the hypothesis that single nucleotide polymorphisms (SNPs) located within genes encoding PD-1 and PD-L1 molecules as well as SNP-SNP interactions (between variations in genes encoding receptor and ligand) are associated with RCC risk and outcomes.

## 2. Results

### 2.1. PDCD1 and PD-L1 Polymorphisms

In the present study, we investigated nine polymorphisms—five of which were located in *PDCD1* gene (encoding PD-1 molecule): rs36084323G>A (PD-1.1), rs11568821G>A (PD-1.3), rs2227981C>T (PD-1.5), rs10204525G>A (PD-1.6), rs7421861T>C and four in *PD-L1* gene: rs822335C>T, rs4143815G>C, rs4742098A>G, rs10815225G>C. Table 1 presents genotype distribution of examined SNPs in ccRCC patients and control subjects. In Table S1 the genotyping results for a group that included patients with a different types of renal cell carcinoma, in addition to ccRCC, are presented.

For all SNPs except three (rs10204525G>A, rs11568821G>A and rs7421861T>C) the genotype distribution was in Hardy-Weinberg Equilibrium (HWE) (Table 1). For rs10204525G>A we found deviation from HWE in controls, resulting from the deficiency of rs10204525 GA heterozygotes (*p* = 0.037, *f* = 0.131). Departures from HWE were also observed for rs7421861T>C and rs11568821G>A polymorphisms in the group of ccRCC patients (*p* = 0.048, *f* = 0.137 and *p* = 0.050, *f* = 0.136, respectively).

### 2.2. Linkage Disequilibrium

The results of linkage disequilibrium (LD) analysis for polymorphisms of *PDCD1* and *PD-L1* are presented in Tables S2 and S3, respectively. As can be seen in these tables, *PDCD1* and *PD-L1* polymorphisms, selected for examination, were not in LD with each other, except for rs4143815G>C and rs4742098A>G in *PD-L1* gene, for which the weak LD was observed (r^2^ = 0.537 and r^2^ = 0.558 for controls and ccRCC patients, respectively) (Table S3).

### 2.3. The Association of PDCD1 and PD-L1 Genes Polymorphisms with ccRCC Risk

Firstly, we observed that two of the nine examined SNPs, mentioned above, seemed to be associated with the risk of clear cell renal cell carcinoma development. Significant difference in genotype distribution between cases and controls was observed inter alia for rs7421861T>C in *PDCD1* (χ^2^ = 6.272; *p* = 0.043). In detail, individuals with rs7421861 TC genotype had 1.5-fold lower risk of ccRCC development in comparison to rs7421861 TT homozygotes (OR = 0.65, CI95% = 0.44; 0.96) while for rare homozygotes (rs7421861 CC) the risk was similar as in the case of individuals with rs7421861 TT genotype (OR = 1.14, CI95% = 0.64; 2.08). Therefore the results suggested the lower risk of ccRCC for rs7421861 TC heterozygotes in comparison to combined group of both homozygotes (TC vs. TT + CC; χ^2^ = 6.058; *p* = 0.014; OR = 0.63, CI95% = 0.43; 0.91).

The second polymorphism for which the association with the risk of ccRCC development was observed was rs10815225G>C in *PD-L1* gene (χ^2^ = 6.981; *p* = 0.031). In the case of this polymorphism, rs10815225 GC genotype was more frequent in the ccRCC patient group as compared to controls (20.5% vs. 13.5%). Therefore, the risk of the disease was more than 1.5-fold higher for individuals being heterozygotes at this polymorphic site (OR = 1.62, CI95% = 0.99; 2.65). Due to the small number of individuals being rs10815225 CC homozygotes (4 individuals with CC genotypes in the control group and 0 among patients), it was difficult to draw any conclusion for this rare genotype. Therefore in the SNP-SNP interaction analysis (presented below) they were combined with GC heterozygotes. No evidence of association between other SNPs investigated here and the risk of ccRCC was noticed.

### 2.4. Haplotype Analysis

The next step was aimed at comparison of *PDCD1* as well as *PD-L1* haplotype frequencies between ccRCC patients and controls. *PD-L1* and *PDCD1* haplotypes with the frequency of more than 3% in both groups are shown respectively in Table 2 and Table S4.

In the case of *PDCD1* gene, we were not able to observe any differences in haplotype frequencies between ccRCC patients and controls; however, such discrepancies were noticed in the case of *PD-L1* gene (global χ^2^ = 21.541; *p* = 0.003). Namely the C C G A (rs822335 rs10815225 rs4143815 rs4742098) haplotype was more frequent in ccRCC patients than in control group (6.3 vs. 3.1%) and increased the risk of developing ccRCC (OR = 2.03, CI95% = 1.07; 3.86). It is worth noting that this haplotype differed from the wild haplotype (most frequent in both groups C G G A) only in the second position corresponding to rs10815225G>C (which was shown to be associated with ccRCC development in our study). The opposite effect—the decreased susceptibility to ccRCC was observed for T G G A (OR = 0.64, CI95% = 0.46; 0.89) as well as for C G C G (OR = 0.55, CI9% = 0.33; 0.93) haplotypes (with common G allele in the second position—rs10815225).

### 2.5. SNP-SNP Interactions between Variations in PDCD1 and PD-L1 Genes in Relation to the ccRCC Risk

Next, we performed the analysis of pairwise SNP-SNP interaction between polymorphisms located in *PDCD1* and *PD-L1* genes, in order to verify the hypothesis that it is not a single SNP, but a combination thereof, which affects the risk of ccRCC. This analysis revealed an interesting relationship–it indicated that the association of rs7421861T>C with ccRCC risk (described above) should be analyzed with the simultaneous consideration of the genotype at rs10815225G>C polymorphic site (and vice versa) (*p* = 0.009) (please see Table S5). It turned out that although the effect of rs7421861 TC genotype was observed in the whole group, this genotype decreased the risk of ccRCC development in individuals with C allele carriers (GC + CC) at rs10815225 polymorphic site (rs7421861 TC vs. TT + CC: χ^2^ = 11.180, *p* = 0.0009, OR = 0.21, CI95% = 0.08; 0.54, in rs10815225 (GC + CC) group) (Table 3). Moreover, in the group of subjects possessing TT or CC genotype at rs7421861 polymorphic site, those who were rs10815225 C allele carriers had higher risk of ccRCC development (GC + CC vs. GG: χ^2^ = 7.306, *p* = 0.007, OR = 2.40, CI95% = 1.25, 4.61) (Table 4).

### 2.6. PDCD1 and PD-L1 Polymorphisms and Overall Survival of ccRCC Patients

According to literature data such clinical parameters as: higher tumor grade, higher stage, presence of sarcomatoid component and presence of venous thrombus are associated with shorter overall survival [11]. In our study we observed the same associations, what confirmed that our group was representative. However, we did not find any associations between variations in *PDCD1* and *PD-L1* genes and overall survival in patients with ccRCC as well as in the group of RCC patients.

## 3. Discussion

The PD-1/PD-L1 axis can be hijacked by tumor cells to avoid anti-tumor immune response. Therefore, genetic polymorphisms which may alter expression or function of PD-1 and PD-L1 molecules have been studied in the context of variety types of cancers. Nonetheless, according to our best knowledge in the literature there is no report regarding renal cell carcinoma. The above mentioned facts prompted us to perform case-control study in order to find possible associations between SNPs in genes encoding molecules from PD-1/PD-L1 axis and ccRCC risk. Based on available literature and in silico analysis, for our research, we selected nine SNPs: five of *PDCD1* gene and four of *PD-L1*. The chosen SNPs cover large linkage disequilibrium (LD) blocks, therefore they may serve as TagSNPs (on the basis of which the conclusion can be drawn for other SNPs included in LD block) (*PDCD1*: rs36084323G>A, rs2227981C>T, rs10204525G>A, *PD-L1*: rs4742098A>G) (Figures S1 and S2). The other ones are located in key regulatory regions (5′ flanking regions or promoters) where transcription factors may bind (*PDCD1*: rs36084323G>A, rs7421861T >, *PD-L1*: rs822335C>T, rs10815225G>C) or in 3′UTR miRNAs binding sites (*PDCD1*: 10204525G>A, PD-L1: rs4143815G>C, rs4742098A>G).

For three polymorphisms of *PDCD1* gene: rs10204525G>A, rs11568821G>A and rs7421861T>C we observed deviation from HWE. As far as rs10204525G>A SNP is concerned the departure was observed in the control group, while in the case of two other SNPs, deviation from HWE were noticed in the patient group. It is noteworthy that genotyping was performed with commercially available assays, and for all SNPs accuracy of genotyping was verified through direct sequencing of randomly selected samples. Since according to literature data [12] in the presence of an association, the deviations from HWE in patients can be treated as a confirmation of the gene-disease association, the results of our HWE analysis may suggest an association of rs11568821G>A and rs7421861T>C with ccRCC risk.

Indeed, from our study came the observation that individuals with rs7421861 TC genotype in *PDCD1* gene had decreased risk of ccRCC development in comparison to individuals possessing homozygous genotypes. The effect of this genetic variant was observed in the whole group of studied individuals (TC vs. TT + CC, OR = 0.63, CI95% = 0.43, 0.91); however the detailed analysis showed that rs7421861 TC genotype decreased risk of ccRCC in rs10815225 C allele carriers (TC vs. TT + CC OR = 0.21, CI95% = 0.08; 0.54). The substantial effect size of rs7421861 TC genotype OR = 0.21 (5-fold decrease) is the reason this effect is observable in the whole group. Recently, Hashemi et al. [13] performed a meta-analysis of studies concerning association between *PDCD1* and *PD-L1* polymorphisms and overall cancer risk. In the case of rs7421861T>C SNP the authors summarized seven studies on different types of cancer and found out that, in contrast to our observation, rs7421861 TC genotype was associated with increased overall cancer risk [13]. However, all the studies included in this meta-analysis were conducted on other than ccRCC types of cancer and on Chinese population with a different genotype distribution at rs7421861 polymorphic site compared to European populations. These arguments could explain the observed discrepancy. Since, according to our best knowledge, our study is the first that analyzed *PDCD1* and *PD-L1* gene polymorphisms in the context of renal cell cancer, further studies will be needed in order to determine whether rs7421861T>C polymorphism may be considered to be a risk factor for this type of cancer. Rs7421861T>C is located in intron 1 of *PDCD1* gene, where potential splicing control components and regulatory elements may be located [14]. Our in silico analysis indicated also the overexpression of PD-1 in ccRCC tissues as compared to control tissues (Figure S3). With regard to our results, it can be supposed that rs7421861 TC genotype that was more frequent in controls, might be associated with decreased expression of PD-1. The association of the heterozygous genotype with the risk of disease may be intriguing; however, in the literature there are articles showing similar relationships [15,16]. Since the polymorphisms of PD-1 and PD-L1 have not been studied in the context of renal cancer so far, our results should be validated in a larger cohort and in other populations to see if such associations exist.

Additionally, in our study we observed the increased risk of ccRCC in case of individuals with rs10815225 GC genotype. Due to the small number of individuals with rs10815225 CC genotype in investigated group it is hard to draw any conclusion regarding this genotype. In the literature there is one report concerning rs10815225G>C SNP in the context of gastric adenocarcinoma [17]; however Tao et al. observed the opposite effect for this polymorphism. Namely the authors noticed lower risk of gastric cancer for individuals with rs10815255 GC as compared to rs10815225 GG [17]. The rs10815225G>C polymorphism was also investigated in the context of colorectal cancer; however, no evidence of association was found by Catalano et al. in Czech population [18]. It would be interesting to verify in further studies whether rs10815225 SNP may be considered to be a risk factor for cancer development and whether the discrepancy between published studies may result from different mechanism underlying these types of cancer. It is worth recalling here that due to the SNP-SNP interaction the effect of rs10815225G>C polymorphism should be considered together with rs7421861T>C.

**Figure 1:**
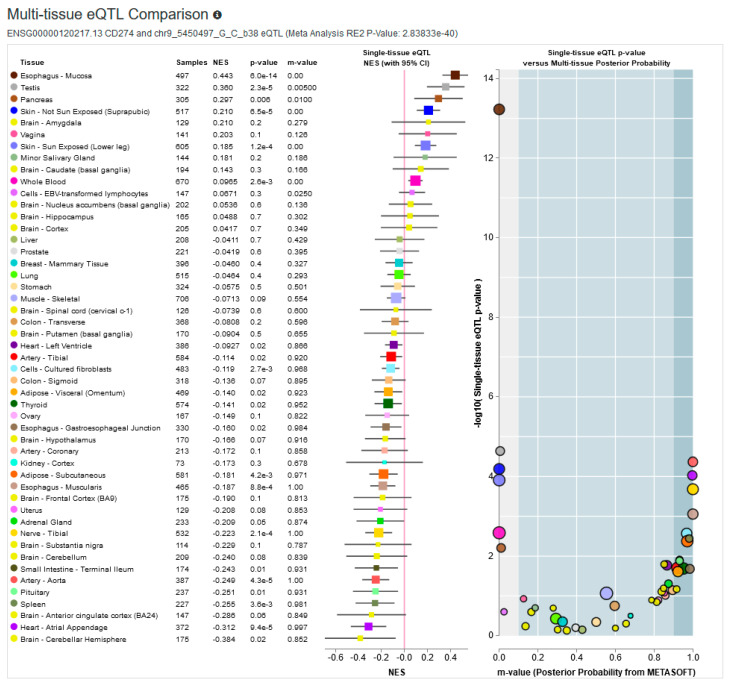
GTEx multi-tissue eQTLs analysis of association between rs10815225 C allele and PD-L1 expression.

Rs10815225G>C is located in the promoter region of *PD-L1* gene. To evaluate global effect of this polymorphism on PD-L1 expression we generated multi-tissue expression quantitative trait locus (eQTL) plot using data from Genotype-Tissue Expression (GTEx) project (Figure 1). The rs10815225 polymorphism seemed to be associated with PD-L1 expression (Meta-Analysis RE2 P-Value: 2.8e-40). The minor allele at rs10815225 polymorphic site (C allele) was associated with higher PD-L1 expression in some tissues e.g., esophagus – mucosa, testis, while for other tissues (e.g., artery-aorta, spleen) the same allele showed association with lower PD-L1 expression. In the case of kidney-cortex, rs10815225 C allele was not associated with PD-L1 expression; however, it is worth noting that only 73 samples were available for this type of tissue. This observation suggests that rs10815225G>C may be associated with tissue-specific regulation of PD-L1 expression.

**Figure 2:**
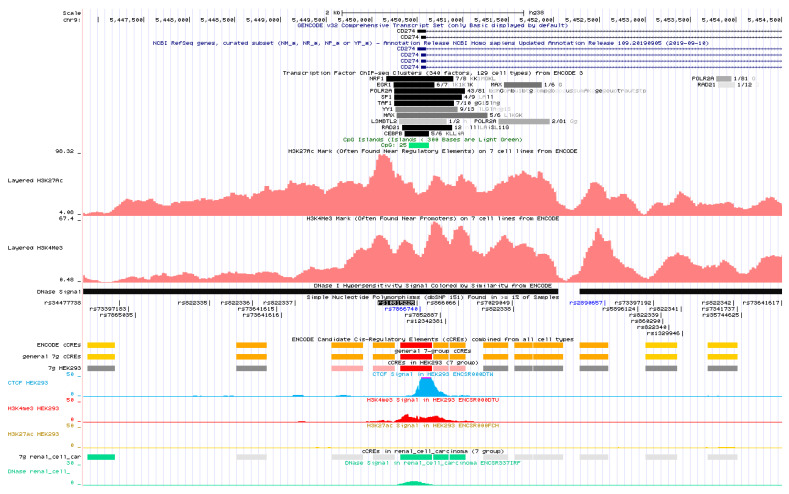
In silico analysis of rs10815225G>C (*PD-L1*) polymorphism in relation to data from ENCODE project. A view of region from UCSC genome browser includes the potential transcription factor binding sites, location of CpG island as well as H3K27Ac, H3K4Me3 and DNase signals on the basis of which the cCREs are classified inter alia into promoter-like and enhancer-like groups etc. Analysis performed for HEK298 cell line and renal cell carcinoma.

In line with that, the in silico analysis of functional relevance of specific gene regions carried out using ENCODE data (Figure 2) indicated that this SNP is located in a candidate cis-regulatory element (cCRE) EH38E2679714 (hg38), which is predicted to have promoter-like signature inter alia in HEK293 cell line (established from primary embryonic human kidney) as well as high DNase signal (characteristics of active gene regulatory elements) in renal cell carcinoma (other data are not available for RCC). As can be seen in Figure 2 rs10815225G>C polymorphism is situated in potential binding site for such transcription factor as SP1 (the SP1 binding motif was predicted not only by ENCODE but also by HaploReg and RegulomeDB bioinformatic tools). Indeed, Tao et al. confirmed (in chromatin immunoprecipitation assay) the binding of SP1 to the promoter region of PD-L1. It was also shown that the examined TF bound to construct with rs10815225 G allele but not to construct with C allele and that the up-regulation or silencing of SP1 resulted respectively in elevated or decreased PD-L1 expression on mRNA and protein level in SGC-7901 cells (gastric cancer cell line). Moreover, Tao et al. described higher mRNA expression of PD-L1 in gastric adenocarcinoma patients with rs10815225 GG genotype than in those with rs10815225 GC genotype. However, it would be worth determining in further studies whether the difference in mRNA expression between individuals with different genotypes at the rs10815225 polymorphic site obtained in gastric cancer patients and that generated from 1000 Genomes (both presented by Tao et al.) should be considered significant. This is important, since Wang et al. [19] raised a doubt about the strength of evidence provided by Tao et al. [17] to prove that elevated level of PD-L1 is run by G-allelic PD-L1. Moreover, the study performed by Wang et al. did not show any difference in expression of PD-L1 (on protein level) between gastric cancer patients with different genotypes at the rs10815225 polymorphic site [19]. The discrepancy between results obtained by Tao et al. [17] and Wang et al. [19] may result from posttranscriptional regulation of PD-L1. However it would be necessary to perform further studies in order to dispel all doubts.

According to the results of our in silico analysis (Figure S3), the elevated level of PD-L1 mRNA was noticed in ccRCC tissues as compared to control tissues. Therefore, it can be assumed that the genotype rs10815225 GC, which were more frequent in ccRCC patients than in controls and increased the risk of ccRCC, might be associated with increased expression of PD-L1. In the context of the results obtained by Tao et al. [17] showing higher risk and increased PD-L1 expression for rs10815225 GG genotype, our observation seems to be unexpected. However, it should be kept in mind that the above mentioned literature data concerned gastric adenocarcinoma, while the subject of our research was renal cell carcinoma. The regulation of PD-L1 expression can be different in the case of these cancers. With regard to in silico analysis (Figure 2), not only SP1, but also other transcription factors (that can be expressed in a tissue-specific manner) can bind, possibly with different affinities for the C and G alleles, to the promoter region where rs10815225 polymorphism is located (e.g., TAF1, EGR1). Taken the above into consideration, it would be interesting to determine in further studies which transcription factors and in what way may regulate PD-L1 expression in the case of ccRCC. It is also worth noting that this SNP is located in CG dinucleotide within CpG island (Figure 2); however a G>C substitution removes one CG dinucleotide but creates another (CCTCCGGGCC). It seems worthwhile to investigate whether the PD-L1 methylation status (and in consequence mRNA expression) may be different depending on the genotype at the rs10815225 polymorphic site.

Interestingly, as was mentioned earlier, the analysis of SNP-SNP interaction (between SNPs in receptor/ligand encoding genes) indicated that polymorphisms rs7421861T>C of *PDCD1* and rs10815225G>C of *PD-L1*, should be considered together in relation to ccRCC risk. To explain the potential biological relevance of detected interaction in relation to ccRCC development, two earlier justified assumptions were made: 1) increased expression of PD-L1 on tumor cells in individuals with C allele in rs10815225 (GC + CC) as compared to those with rs10815225 GG and 2) decreased expression of PD-1 on T cells in individuals with rs7421861 TC genotype in comparison to subjects with rs7421861 TT or CC genotype.

As described above, our analysis showed that the C allele in rs10815225 (GC or CC genotype) increased (more than twice, OR = 2.4) the risk of ccRCC development in individuals with rs7421861 TT or CC genotype (Table 4). Taken our assumptions into account, individuals with such combination of genotypes would have elevated level of both molecules – PD-L1 on tumor cells and PD-1 on T cells. Therefore, it can be supposed that in the analyzed case, anti-tumor response would be inhibited by the PD-1/PD-L1 axis and this would generate microenvironment facilitating tumor growth and invasion. This mechanism would explain the increased risk of developing ccRCC in this group of individuals.

In contrast, rs7421861 TC genotype was associated with lower risk of ccRCC development in rs10815225 C allele carriers (Table 3). According to our assumptions, increased level of PD-L1 on tumor cells and low level of PD-1 on T cell would be observed in individuals with such genotypes at analyzed polymorphic sites. Due to the limited level of PD-1 receptor on T cells, even in case of overexpression of the ligand—PD-L1, anti-tumor response would not be inhibited and that would explain the observed lower risk of ccRCC development.

In the next step it would be necessary to investigate PD-1 and PD-L1 expression (on mRNA and protein level) in relation to genotype at rs7421861 and rs10815225 polymorphic sites, respectively, in order to verify that the assumptions described above can be considered true.

We have also analyzed available clinical data and obtained genetic data in relation to the long-term survival (6.5 year). We have observed that factors commonly associated with poor prognosis [11] showed associations also in our group of patients what confirmed that examined by us population was representative for a typical renal cancer population. However, none of the investigated here SNPs neither in *PDCD1* nor in *PD-L1* gene were associated with overall survival in RCC group and ccRCC subgroup of patients.

Our study revealed that genetic variants of *PDCD1* and *PD-L1* may be associated with the risk of ccRCC development and that in association studies it is worth considering the interactions between variants in the genes encoding molecules belonging to the receptor/ligand axis. Since this is the first study of *PDCD1* and *PD-L1* polymorphisms in the context of ccRCC, we believe that our research will broaden current knowledge of the genetic basis of this type of cancer.

## 4. Materials and Methods

### 4.1. Subjects

A total of 237 renal cell carcinoma patients (82 females and 155 males) diagnosed in the Department of Urology and Oncologic Urology at Wroclaw Medical University, a tertiary urologic cancer center were included in the present study. Most of them (N = 208) suffered from the clear cell renal cell carcinoma. The detailed characteristic of patients is presented in Table 5. Patients were recruited for the previous project (on the basis of approval of the Ethics Committee of the Wroclaw Medical University (KB 55/2010)). Written informed consent was obtained from all participants. Retrospective study and reuse of the material was approved by the Ethics Committee of the Wroclaw Medical University (KB 587/2020). The control population was consisted of 256 blood donors of Polish Caucasian origin (112 females and 144 males).

### 4.2. Genotyping

DNA from each individual was isolated from venous blood using the QIAamp DNA Blood Mini Kit (Qiagen, Hilden, Germany) according to the manufacturer’s protocol. The following single nucleotide polymorphisms (SNPs) in *PDCD1* gene: rs36084323G>A (PD-1.1), rs11568821G>A (PD-1.3), rs2227981C>T (PD-1.5), rs10204525G>A (PD-1.6), rs7421861T>C as well as in *PD-L1* gene: rs822335C>T, rs4143815G>C, rs4742098A>G, rs10815225G>C—were genotyped with the use of appropriate TaqMan SNP Genotyping Assays (in detail presented in Table S6). For all SNPs, the accuracy of genotyping for randomly selected samples was verified by direct Sanger sequencing.

### 4.3. In silico Analysis

To perform in silico analysis and predict possible functional effects for selected SNPs the following tools were applied:

(1) ENCODE: Encyclopedia of DNA Elements [20,21]
(2) Genome Browser [22,23,24]
(3) Genotype-Tissue Expression Portal (GTEx) (V8) [25]
(4) HaploReg v4.1 (HaploReg was also applied to generate LD blocks for investigated SNPs) [26,27]
(5) RegulomeDB [28]

### 4.4. Statistical Analysis

To test hypothesis that two groups (patients and controls) have the same distribution of genotype counts chi-square, χ2, was applied. Odds ratio (OR) and confidence intervals for OR at 1 − α = 0.95 (CI 95%) were computed as the measures of effect size. Measure of deviation from HWE was $f=\frac{p_{cc}-p_{c}^{2}}{p_{c}(1-p_{c})}$ where *P_c_* and *P_cc_* correspond to frequencies of allele *C* and genotype *CC*. *f* < 0 in case of deficiency of homozygotes, *f* > 0 corresponds to deficiency of heterozygotes and *f* = 0 when locus is in Hardy-Weinberg equilibrium. Linkage equilibrium analysis (r^2^) and haplotype frequencies were estimated with application of SHEsis software [29]. Overall survival with different clinicopathological variables was calculated using Kaplan-Meier analysis. A log-rank test was used to assess the association between predictor variables and patient survival. A Cox proportional hazards model was used to assess the impact of a range of qualitative and continuous variables on survival.

## 5. Conclusions

In conclusion, the results of our study suggest that polymorphisms of PD-1/PD-L1 axis genes may be associated with the risk of ccRCC development. Since PD-1 and PD-L1 genetic variants have not been examined in the context of ccRCC so far, further studies are necessary to validate observed by us associations. The next key step that must be taken is to quantify the expression of PD-1 and PD-L1 (on mRNA and protein level) in relation to rs7421861 and rs10815225 genotype, respectively. It would also be interesting to investigate the possible effect of rs7421861 SNP on the level of PD-1 isoforms (generated by alternative splicing), as well as the effect of rs10815225 polymorphism on PD-L1 methylation status.

Importantly, our research showed that in order to find possible risk factors for renal cell carcinoma (and possibly other types of cancer), it is worth investigating SNP-SNP interactions between variants in the genes encoding the receptor/ligand. This could be an important clue that should be considered in future studies on polymorphisms in genes encoding immune checkpoint molecules in the context of cancer risk development. With regard to the fact that as far as we know in the literature there is no published data regarding associations of *PDCD1* and *PD-L1* polymorphisms with ccRCC risk and outcomes, we believe that our research will broaden current knowledge of the genetic basis of this type of cancer.

## Figures and Tables

**Table 1:** Genotype distribution of investigated *PDCD1* and *PD-L1* polymorphisms in clear cell renal cell carcinoma (ccRCC) patients and controls.

Gene	Polymorphisms	Genotype	ccRCC Patients ^a^			Controls ^b^			OR (CI95%)	ccRCC vs. Controls
			N	%	HWE	N	%	HWE		
***PDCD1 (PD-1)*** ***(-strand)***	rs36084323 (PD-1.1)	GG	201	96.6	*p* = 0.805; *f* = −0.017	249	97.3	*p* = 0.824; *f* = −0.014	1 ^c^	χ^2^ = 0.156; *p* = 0.693
		GA	7	3.4		7	2.7		1.24 (0.44; 3.46)	
		AA	0	0.00		0	0.00		−	
	rs11568821 (PD-1.3)	GG	164	78.8	*p* = 0.050; *f* = 0.136	206	80.5	*p* = 0.445; *f* = 0.048	1 ^c^	χ^2^ = 0.974; *p* = 0.614
		GA	38	18.3		46	18.0		1.04 (0.65; 1.67)	
		AA	6	2.9		4	1.6		1.81 (0.54; 6.14)	
	rs2227981 (PD-1.5)	CC	70	33.7	*p* = 0.587; *f* = 0.038	82	32.0	*p* = 0.361; *f* = −0.057	1 ^c^	χ^2^ = 1.068; *p* = 0.586
		CT	98	47.1		132	51.6		0.87 (0.58; 1.31)	
		TT	40	19.2		42	16.4		1.12 (0.65; 1.90)	
	rs10204525 (PD-1.6)	GG	172	82.7	*p* = 0.581; *f* = −0.038	208	81.2	*p* = 0.037; *f* = 0.131	1 ^c^	χ^2^ = 2.681; *p* = 0.262
		GA	35	16.8		42	16.4		1.01 (0.62; 1.65)	
		AA	1	0.5		6	2.3		0.28 (0.05; 1.67)	
	rs7421861	TT	96	46.2	*p* = 0.048; *f* = 0.137	99	38.7	*p* = 0.142; *f* = −0.052	**1 ^c^**	**χ^2^ = 6.272;** ***p* = 0.043**
		TC	81	38.9		129	50.4		**0.65 (0.44; 0.96)**	
		CC	31	14.9		28	10.9		**1.14 (0.64; 2.08)**	
***PD-L1*** **(+strand)**	PD-L1 (rs822335)	CC	77	37.6	*p* = 0.149; *f* = −0.101	102	40.5	*p* = 0.429; *f* = 0.050	1 ^c^	χ^2^ = 2.600; *p* = 0.273
		CT	105	51.2		112	44.4		1.24 (0.83; 1.84)	
		TT	23	11.2		38	15.1		0.81 (0.45; 1.46)	
	PD-L1 (rs4143815)	GG	97	47.3	*p* = 0.193; *f* = 0.091	122	48.4	*p* = 0.455; *f* = 0.047	1 ^c^	χ^2^ = 0.427; *p* = 0.808
		GC	82	40.0		103	40.9		1.00 (0.68; 1.48)	
		CC	26	12.7		27	10.7		1.21 (0.67; 2.20)	
	PD-L1 (rs4742098)	AA	122	59.5	*p* = 0.697; *f* = 0.027	137	54.4	*p* = 0.737; *f* = −0.021	1 ^c^	χ^2^ = 1.231; *p* = 0.540
		AG	71	34.6		99	39.3		0.81 (0.55; 1.19)	
		GG	12	5.9		16	6.3		0.85 (0.39; 1.84)	
	PD-L1 (rs10815225)	GG	163	79.5	*p* = 0.102; *f* = −0.114	214	84.9	*p* = 0.064; *f* = 0.117	**1 ^c^**	**χ^2^ = 6.981;** ***p* = 0.030**
		GC	42	20.5		34	13.5		**1.62 (0.99; 2.65)**	
		CC	0	0.0		4	1.6		**0.15 (0.01; 2.73)**	

^a^ N = 208 for *PDCD1* and N = 205 for *PD-L1* SNPs; ^b^ N = 256 for *PDCD1* and N = 252 for *PD-L1* SNPs; ^c^ reference group; OR, odds ratio; CI, confidence intervals; HWE, Test for Hardy-Weinberg equilibrium; *f*, departure from HWE. Significant results were bolded.

**Table 2:** *PD-L1* haplotype analysis in relation to ccRCC risk.

*PD-L1* Haplotype				ccRCC Patients N (%)	Controls N (%)	χ^2^	*p*	OR (CI95%)
rs822335	rs10815225	rs4143815	rs4742098					
C	G	C	A	14.5 (3.5)	20.3 (4.0)	0.205	0.651	0.85 (0.43; 1.70)
C	C	C	G	15.4 (3.7)	22.3 (4.4)	0.346	0.557	0.82 (0.42; 1.59)
**C**	**G**	**C**	**G**	**22.7 (5.5)**	**47.3 (9.4)**	**5.154**	**0.023**	**0.55** **(0.33; 0.93)**
**C**	**C**	**G**	**A**	**25.8 (6.3)**	**15.8 (3.1)**	**4.873**	**0.027**	**2.03** **(1.07; 3.86)**
C	G	G	A	177.5 (43.3)	197.2 (39.1)	1.003	0.317	1.15 (0.88; 1.50)
T	G	C	A	27.8 (6.8)	20.9 (4.1)	2.857	0.091	1.65 (0.92; 2.95)
T	G	C	G	53.6 (13.1)	46.2 (9.2)	3.107	0.078	1.45 (0.96; 2.21)
**T**	**G**	**G**	**A**	**69.3 (16.9)**	**118.8 (23.6)**	**7.051**	**0.008**	**0.64** **(0.46; 0.89)**
				**global χ^2^ = 21.541; *p* = 0.003**				

OR, odds ratio; CI, confidence intervals. Significant results were bolded.

**Table 3:** Genotype distribution of rs7421861T>C polymorphism in rs10815225 GG and rs10815225 GC + CC individuals.

		rs10815225 GG				rs10815225 GC + CC			
		ccRCC Patients	Controls	OR (CI95%)		ccRCC Patients	Controls	OR (CI95%)	
**rs7421861**	**TT + CC**	92 (56.4%)	109 (50.9%)	1 ^a^	χ^2^ = 1.124; *p* = 0.289	33 (78.6)	16 (42.1%)	1 ^a^	**χ^2^ = 11.038;** ***p* = 0.0009**
	**TC**	71 (43.6%)	105 (49.0%)	0.80 (0.53; 1.31)		9 (21.4%)	22 (52.4%)	**0.21 (0.08; 0.54)**	

^a^ reference group; OR, odds ratio; CI, confidence intervals. Significant results were bolded.

**Table 4:** Genotype distribution of rs10815225G>C polymorphism in rs7421861 TT + CC and rs7421861 TC individuals.

		rs7421861 TT + CC				rs7421861 TC			
		ccRCC Patients	Controls	OR (CI95%)		ccRCC Patients	Controls	OR (CI95%)	
**rs10815225**	**GG**	92 (73.6%)	109 (87.2%)	1 ^a^	**χ^2^ = 7.306;** ***p* = 0.007**	71 (88.7%)	105 (82.7%)	1 ^a^	χ^2^ = 1.415; *p* = 0.234
	**GC + CC**	33 (26.4%)	16 (12.8%)	**2.40 (1.25; 4.61)**		9 (11.3%)	22 (17.3%)	0.62 (0.28; 1.41)	

^a^ reference group; OR, odds ratio; CI, confidence intervals. Significant results were bolded.

**Table 5:** Characteristics of RCC and ccRCC patients.

	RCC	ccRCC
**Number of patients**	237	208
**Gender**		
Male	155 (65.4%)	135 (64.9%)
Female	82 (34.6)	73 (35.1%)
**Age at diagnosis**		
Q1 (years)	57	57
Median (years)	62	62
Q3 (years)	71	70.75
Range (min-max) (years)	21–85	21–85
**Stage at presentation**		
I	114 (48.1%)	101 (48.6%)
II	31 (13.1%)	26 (12.5%)
III	26 (11.0%)	24 (11.5%)
IV	66 (27.8%)	57 (27.4%)
**Grade**		
I	105 (44.3%)	96 (46.2%)
II	64 (27.0%)	62 (29.8%)
III	36 (15.2%)	35 (16.8%)
IV	9 (3.8%)	9 (4.3%)
n.a. ^a^	23 (9.7%)	6 (2.9%)
**Presence of venous thrombus**		
absent	218 (92.0%)	193 (92.8%)
present	19 (8.0%)	15 (7.2%)
**Presence of sarcomatoid component**		
absent	203 (85.7)	182 (87.5%)
present	9 (3.8%)	9 (4.3%)
n.a. ^a^	25 (10.5%)	17 (8.2%)
**Survival status**		
Dead	117 (49.4%)	105 (50.5%)
Alive	120 (50.6%)	103 (49.5%)

^a^ n.a.—data not available.

## Supplementary Materials

The following are available online at https://www.mdpi.com/2072-6694/12/12/3521/s1, Table S1: Genotype distribution of investigated *PDCD1* and *PD-L1* polymorphisms in renal cell carcinoma (RCC) patients and controls. Table S2: Analysis of linkage disequilibrium (r^2^) between genetic variants located in *PDCD1* gene. Table S3: Analysis of linkage disequilibrium (r^2^) between genetic variants located in *PD-L1* gene. Table S4: *PDCD1* haplotype analysis in relation to ccRCC risk. Table S5. Rs7421861 × rs10815225 interaction-observed and expected genotype distributions in ccRCC patients and control subjects. Table S6. List of Assays used in the study to perform genotyping of examined SNP. Figure S1: Linkage disequilibrium (LD) blocks generated with application of HaploReg v4.1 presenting SNPs being in strong LD (r^2^ > 0.8) with (A) rs36084323G>A (PD-1.1), (B) rs11568821G>A (PD-1.3), (C) rs2227981C>T (PD-1.5), (D) rs10204525G>A (PD-1.6), (E) rs7421861T>C. Figure S2: Linkage disequilibrium (LD) blocks generated with application of HaploReg v4.1 presenting SNPs being in strong LD (r^2^ > 0.8) with rs822335C>T, rs4143815G>C, rs4742098A>G, rs10815225G>C. Figure S3: PDCD1 and PD-L1 overexpression in ccRCC tissues as compared to control tissues.
