# Supplementary material for: SNP-SNP Interaction in Genes Encoding PD-1/PD-L1 Axis as a Potential Risk Factor for Clear Cell Renal Cell Carcinoma
Source: Cancers (Basel). 2020 Nov 26;12(12):3521. doi: 10.3390/cancers12123521 (PMC7760680; doi:10.3390/cancers12123521)
Supplement: Supplementary file 1 [file cancers-12-03521-s001.pdf]

cancers

Supplementary Materials

SNP-SNP Interaction in Genes Encoding PD-1/PD-L1 Axis as a Potential Risk Factor for Clear Cell Renal Cell Carcinoma

Marta Wagner, Krzysztof Tupikowski, Monika Jasek, Anna Tomkiewicz, Agata Witkowicz, Kuba Ptaszkowski, Pawel Karpinski, Romuald Zdrojowy, Agnieszka Halon and Lidia Karabon

Table S1. Genotype distribution of investigated *PDCD1* and *PD-L1* polymorphisms in Renal Cell Carcinoma (RCC) patients and controls.

Gene	Polymorphisms	Genotype	RCC patients (N = 237)			Controls (N = 256)			OR (CI95%)	*p*
			N	%	HWE	N	%	HWE		
PDCD1 (PD-1)	rs36084323 (PD-1.1)	GG	229	96.6	$p = 0.070$; $f = -0.017$	249	97.3	$p = 0.824$; $f = -0.014$	1	$\chi^2 = 0.171$; $p = 0.679$
		GA	8	3.4		7	2.7		1.23 (0.45; 3.34)	
		AA	0	0.0		0	0.00		–	
	rs11568821 (PD-1.3)	GG	188<br>43	79.3	$p = 0.075$; $f = 0.116$	206	80.5	$p = 0.445$; $f = 0.048$	1	$\chi^2 = 0.592$; $p = 0.744$
		GA	43	18.1		46	18.0		1.02 (0.65; 1.62)	
		AA	6	2.5		4	1.6		1.58 (0.47; 5.35)	
	rs2227981 (PD-1.5)	CC	80	33.8	$p = 0.713$; $f = 0.024$	82	32.0	$p = 0.361$; $f = -0.057$	1	$\chi^2 = 0.814$; $p = 0.666$
		CT	113	47.7		132	51.6		0.88 (0.59; 1.30)	
		TT	44	18.6		42	16.4		1.07 (0.64; 1.81)	
	rs10204525 (PD-1.6)	GG	195	82.3	$p = 0.454$; $f = -0.049$	208	81.2	$p = 0.037$; $f = 0.131$	1	$\chi^2 = 3.275$; $p = 0.194$
		GA	41	17.3		42	16.4		1.04 (0.65; 1.47)	
		AA	1	1		6	2.3		0.25 (0.04; 1.47)	
	rs7421861	TT	107	45.1	$p = 0.072$; $f = 0.117$	99	38.7	$p = 0.142$; $f = -0.052$	1	$\chi^2 = 5.325$; $p = 0.063$
		TC	95	40.1		129	50.4		0.68 (0.47; 1.00)	
		CC	35	14.8		28	10.9		1.15 (0.66; 2.03)	
PD-L1	PD-L1 (rs822335)	CC	84	35.9	$p = 0.105$; $f = -0.106$	102	40.5	$p = 0.429$; $f = 0.050$	1	$\chi^2 = 3.022$; $p = 0.221$
		CT	122	52.1		112	44.4		1.32 (0.90; 1.94)	
		TT	28	12.0		38	15.1		0.90 (0.51; 1.58)	
	PD-L1 (rs4143815)	GG	107	45.7	$p = 0.282$; $f = 0.070$	122	48.41	$p = 0.455$; $f = 0.047$	1	$\chi^2 = 0.655$; $p = 0.721$
		GC	97	41.5		103	40.88		1.07 (0.73; 1.57)	
		CC	30	12.8		27	10.71		1.26 (0.71; 2.25)	
	PD-L1 (rs4742098)	AA	136	58.1	$p = 0.628$; $f = 0.032$	137	54.4	$p = 0.737$; $f = -0.021$	1	$\chi^2 = 0.777$; $p = 0.678$
		AG	83	35.3		99	39.3		0.85 (0.58; 1.23)	
		GG	15	6.4		16	6.3		0.95 (0.45; 1.97)	
	PD-L1 (rs10815225)	GG	185	79.1	$p = 0.941$; $f = 0.005$	214	84.92	$p = 0.064$; $f = 0.117$	1	$\chi^2 = 3.389$; $p = 0.184$
		GC	46	19.7		34	13.49		1.56 (0.96; 2.52)	
		CC	3	1.3		4	1.59		0.90 (0.22; 3.69)	

cancers

MDPI

Table S2. Analysis of linkage disequilibrium (r^2) between genetic variants located in *PDCD1* gene.

**Controls**				
	**rs11568821**	**rs2227981**	**rs10204525**	**rs7421861**
**rs36084323**	0.001	0.010	0.118	0.000
**rs11568821**	–	0.057	0.004	0.176
**rs2227981**	–	–	0.082	0.372
**rs10204525**	–	–	–	0.053
**ccRCC patients**				
	**rs11568821**	**rs2227981**	**rs10204525**	**rs7421861**
**rs36084323**	0.002	0.012	0.120	0.009
**rs11568821**	–	0.102	0.013	0.242
**rs2227981**	–	–	0.064	0.343
**rs10204525**	–	–	–	0.051

cancers

MDPI

Table S3. Analysis of linkage disequilibrium (r^2) between genetic variants located in *PD-L1* gene.

Controls			
	**rs4143815**	**rs4742098**	**rs10815225**
**rs822335**	0.006	0.000	0.054
**rs4143815**	–	0.537	0.019
**rs4742098**	–	–	0.064
**ccRCC patients**			
	**rs4143815**	**rs4742098**	**rs10815225**
**rs822335**	0.125	0.058	0.066
**rs4143815**	–	0.558	0.003
**rs4742098**	–	–	0.021

Table S4. *PDCD1* haplotype analysis in relation to ccRCC risk.

PDCD1 haplotype					ccRCC patients N (%)	Controls N (%)	χ^2	*p*	OR (CI95%)
**rs36084323**	**rs7421861**	**rs11568821**	**rs2227981**	**rs10204525**					
G	C	A	C	C	48.7 (11.7)	51.1 (10.0)	0.750	0.386	1.20 (0.79; 1.82)
G	T	G	C	C	89.4 (21.5)	106.1 (20.7)	0.096	0.757	1.05 (0.76; 1.44)
G	C	G	C	C	90.0 (21.6)	130.1 (25.4)	1.749	0.186	0.81 (0.60; 1.11)
G	T	G	T	C	145.3 (34.9)	166.2 (32.5)	0.696	0.404	1.12 (0.85; 1.48)
G	T	G	T	T	28.8 (6.9)	43.1 (8.4)	0.706	0.401	0.81 (0.50; 1.32)
						**global χ^2 = 3.167; *p* = 0.530**			

Significant results were bolded

cancers

MDPI

Table S5. Rs7421861 x rs10815225 interaction - observed and expected genotype distributions in ccRCC patients and control subjects.

**ccRCC patients**											
**Observed**					**Expected**					χ^2	p
**Genotype**		**rs7421861**			**Genotype**		**rs7421861**				
		**TT+CC**	**TC**	**∑**			**TT+CC**	**TC**	**∑**		
**rs10815225**	**GG**	92	71	163	**rs10815225**	**GG**	99.39	63.61	163.0	**6.873**	**0.009**
	**GC+CC**	33	9	42		**GC**	25.61	16.39	42.0		
	**∑**	125	80	205		**∑**	125.0	80.0	205.0		
**Controls**											
**Observed**					**Expected**					χ^2	p
**Genotype**		**rs7421861**			**Genotype**		**rs7421861**				
		**TT+CC**	**TC**	**∑**			**TT+CC**	**TC**	**∑**		
**rs10815225**	**GG**	109	105	214	**rs10815225**	**GG**	106.15	107.85	214.0	1.006	0.316
	**GC+CC**	16	22	38		**GC+CC**	18.85	19.15	38.0		
	**∑**	125	127	252		**∑**	125	127	252.0		

In ccRCC patient group there is a difference between observed and expected number of genotypes (p = 0.009). while in controls the observed number of genotypes did not differ from the expected (p = 0.316). Significant results were bolded.

cancers

MDPI

Table S6. List of Assays used in the study to perform genotyping of examined SNP.

Gene	Polymorphism	Assay ID
	rs36084323G>A	C__57931321_10
	rs7421861T>C	C__26891639_10
PDCD1	rs11568821G>A	C__57931290_10
	rs2227981C>T	C__57931286_20
	rs10204525G>A	C____172862_20
	rs10815225 G>C	C___1348557_30
PD-L1	rs822335 C>T	C___7590674_10
	rs4143815 G>C	C__31941235_10
	rs4742098 A>G	C__27957750_10

cancers

MDPI

A

Query SNP: rs36084323 and variants with r^2 >= 0.8

chr	pos (hg38)	LD (r^2)	LD (D')	variant	Ref	Alt	AFR freq	AMR freq	ASN freq	EUR freq	SiPhy cons	Promoter histone marks	Enhancer histone marks	DNAse	Proteins bound	Motifs changed	NHGRI/EBI GWAS hits	GRASP QTL hits	Selected eQTL hits	GENCODE genes	dbSNP func annot
2	241812307	0.82	1	rs76182246	T	G	0.00	0.18	0.46	0.01			4 tissues	LNG		4 altered motifs				NEU4	intronic
2	241814177	0.82	1	rs4675992	C	G	0.00	0.18	0.48	0.01			8 tissues			6 altered motifs				NEU4	intronic
2	241818736	0.82	1	rs2178827	C	G	0.00	0.18	0.48	0.01		LIV	6 tissues	LIV		9 altered motifs				1.3kb 3' of NEU4	
2	241822233	0.82	1	rs75754715	T	C	0.01	0.18	0.47	0.01			9 tissues			BCL,YY1,Zfx				5.7kb 3' of NEU4	
2	241822298	0.82	1	rs80184609	G	C	0.01	0.18	0.47	0.01			9 tissues			7 altered motifs				5.7kb 3' of NEU4	
2	241822909	0.82	1	rs183584878	C	G	0.00	0.18	0.46	0.01		BRST	KID, THYM							6.4kb 3' of NEU4	
2	241830968	0.82	1	rs34931532	C	T	0.00	0.18	0.47	0.01						Irf				13kb 5' of AC114730.2	
2	241833979	0.82	1	rs35471993	A	G	0.01	0.18	0.47	0.01		BLD, BONE	ESDR, ADRL			ATF3,CTCF,RXRA				10kb 5' of AC114730.2	
2	241836300	0.82	1	rs76285602	C	T	0.00	0.18	0.47	0.01					CTCF	4 altered motifs				8.1kb 5' of AC114730.2	
2	241842121	0.82	1	rs4675957	G	T	0.00	0.18	0.47	0.01			4 tissues	IPSC,BLD,BLD						2.3kb 5' of AC114730.2	
2	241845073	0.82	1	rs77227651	G	T	0.00	0.18	0.47	0.01		5 tissues	16 tissues			4 altered motifs				36bp 3' of AC114730.2	
2	241845422	1	1	rs76840685	A	C	0.00	0.19	0.47	0.01		BLD, FAT, MUS	12 tissues	BLD,LNG,BLD		4 altered motifs				385bp 3' of AC114730.2	
2	241847314	1	1	rs72484064	G	A	0.00	0.19	0.47	0.01						6 altered motifs				2.3kb 3' of AC114730.2	
2	241849417	1	1	rs28699177	C	T	0.01	0.19	0.47	0.01			4 tissues			COMP1,CTCF,RP58				463bp 3' of PDCD1	
2	241849525	1	1	rs28542728	G	A	0.00	0.19	0.47	0.01			5 tissues							355bp 3' of PDCD1	
2	241851281	1	1	rs2227982	G	A	0.00	0.19	0.47	0.01			IPSC			PTF1-beta				PDCD1	missense
2	241852468	1	1	rs34819629	C	T	0.00	0.19	0.47	0.01			ESDR, IPSC, MUS			ERalpha-a				PDCD1	intronic
2	241855150	0.92	1	rs80166521	C	T	0.01	0.19	0.47	0.02			8 tissues	BLD,HRT,LNG		4 altered motifs				PDCD1	intronic
2	241857110	1	1	rs41509544	G	A	0.13	0.20	0.48	0.01		BLD, GI	5 tissues	BLD,LIV						PDCD1	intronic
2	241857532	1	1	rs202017368	T	TC	0.01	0.19	0.49	0.01		4 tissues	BLD, SKIN, SPLN			4 altered motifs				PDCD1	intronic
2	241857537	1	1	rs142194983	C	CA	0.01	0.19	0.49	0.01		4 tissues	BLD, SKIN, SPLN			5 altered motifs				PDCD1	intronic
2	241857786	1	1	rs75565781	G	C	0.01	0.19	0.49	0.01		4 tissues	SKIN, SPLN			ERalpha-a,Rad21,SIX5				PDCD1	intronic
2	241857939	0.91	1	rs143627472	19-mer	T	0.05	0.18	0.46	0.01		4 tissues	4 tissues			6 altered motifs				PDCD1	intronic
2	241857978	0.91	1	rs74000562	C	T	0.07	0.17	0.45	0.01		4 tissues	4 tissues			4 altered motifs				PDCD1	intronic
2	241857996	0.82	0.91	rs74000563	T	G	0.06	0.18	0.45	0.01		4 tissues	4 tissues			Foxa,GR				PDCD1	intronic
2	241858014	0.91	1	rs74000564	T	C	0.04	0.18	0.45	0.01		4 tissues	4 tissues			9 altered motifs				PDCD1	intronic
2	241858082	1	1	rs74000565	C	T	0.04	0.19	0.49	0.01		4 tissues	BLD, SPLN, LIV	BLD		CACD,SP1,SRF				PDCD1	intronic
2	241858748	1	1	rs35933396	G	A	0.04	0.19	0.49	0.01		5 tissues	11 tissues	4 tissues	4 bound proteins	NF-E2,Pou2f2				PDCD1	intronic
2	241859444	1	1	rs36084323	C	T	0.04	0.19	0.48	0.01		5 tissues	16 tissues	MUS,THYM,BLD	CTCF,HSF1	4 altered motifs				535bp 5' of PDCD1	
2	241860237	1	1	rs41360545	T	C	0.04	0.19	0.49	0.01		9 tissues	14 tissues	6 tissues	CTCF,EBF1	8 altered motifs				1.3kb 5' of PDCD1	
2	241860758	0.84	1	rs28394424	A	G	0.02	0.19	0.49	0.02		BLD, MUS	14 tissues			6 altered motifs				1.8kb 5' of PDCD1	
2	241865936	0.84	1	rs199959525	TGTG	T	0.00	0.17	0.48	0.02		4 tissues	8 tissues	7 tissues	TR4,CMYC	Ets,Pax-4,RREB-1				3.7kb 5' of CXXC11	
2	241866923	0.84	1	rs75247106	C	T	0.00	0.19	0.49	0.02		THYM	9 tissues	6 tissues		AP-2,SP1				2.7kb 5' of CXXC11	
2	241867868	0.84	1	rs80336268	G	A	0.00	0.19	0.48	0.02		BLD, THYM, SPLN	6 tissues			BCL,Pax-5,Spz1				1.7kb 5' of CXXC11	
2	241868458	0.84	1	rs78051760	C	T	0.00	0.19	0.49	0.02		BLD, THYM	13 tissues	4 tissues		Znf143				1.1kb 5' of CXXC11	
2	241868480	1	1	rs77635907	A	G	0.00	0.19	0.49	0.01		BLD, THYM	13 tissues	4 tissues		7 altered motifs				1.1kb 5' of CXXC11	
2	241868548	0.84	1	rs79851648	C	T	0.00	0.19	0.49	0.02		BLD, THYM	13 tissues	4 tissues		CEBPB,Hsf,STAT				1.1kb 5' of CXXC11	
2	241869143	0.84	1	rs6742353	T	C	0.08	0.19	0.49	0.02			7 tissues			Pax-4				456bp 5' of CXXC11	intronic
2	241869518	0.84	1	rs28486065	C	G	0.00	0.19	0.49	0.02		BRN	6 tissues	BLD		11 altered motifs				81bp 5' of CXXC11	intronic
2	241869714	0.84	1	rs75506522	G	C	0.01	0.18	0.49	0.02		BRN	7 tissues	BRN		21 altered motifs				CXXC11	intronic
2	241875612	0.84	1	rs28564919	C	T	0.17	0.20	0.48	0.02			BLD							1.8kb 3' of CXXC11	

B

cancers

MDPI

Query SNP: rs11568821 and variants with r^2 >= 0.8

chr	pos (hg38)	LD (r^2)	LD (D')	variant	Ref	Alt	AFR freq	AMR freq	ASN freq	EUR freq	SiPhy cons	Promoter histone marks	Enhancer histone marks	DNAse	Proteins bound	Motifs changed	NHGRI/EBI GWAS hits	GRASP QTL hits	Selected eQTL hits	GENCODE genes	dbSNP func annot
2	241824526	0.85	0.95	rs200789545	7-mer	A	0.18	0.07	0.00	0.10			BLD			5 altered motifs			8 hits	8kb 3' of NEU4	
2	241824537	0.85	0.95	rs202244286	TTCTTC	T	0.18	0.07	0.00	0.10			BLD			Irf,TATA,p300			8 hits	8kb 3' of NEU4	
2	241833477	0.9	0.97	rs74898709	G	A	0.16	0.07	0.00	0.10		STRM, BLD	8 tissues	8 tissues	CFOS	CEBPB,Egr-1,Gmeb1			9 hits	11kb 5' of AC114730.2	
2	241839789	0.94	0.97	rs79388822	T	C	0.01	0.06	0.00	0.10						Homez,Mef2,Zfp105			10 hits	4.6kb 5' of AC114730.2	
2	241842504	0.97	0.99	rs111233827	G	A	0.01	0.06	0.00	0.10			5 tissues						10 hits	1.9kb 5' of AC114730.2	
2	241851760	1	1	rs11568821	C	T	0.01	0.06	0.00	0.10			ESDR, IPSC			7 altered motifs			10 hits	PDCD1	intronic
2	241864233	0.89	0.96	rs76768583	T	C	0.01	0.06	0.00	0.10		BLD, STRM	8 tissues	BLD		8 altered motifs			9 hits	5.3kb 5' of PDCD1	
2	241875344	0.85	0.96	rs77803400	A	G	0.02	0.10	0.06	0.11			BLD			Irf,p300			9 hits	1.5kb 3' of CXXC11	

C

Query SNP: rs2227981 and variants with r^2 >= 0.8

chr	pos (hg38)	LD (r^2)	LD (D')	variant	Ref	Alt	AFR freq	AMR freq	ASN freq	EUR freq	SiPhy cons	Promoter histone marks	Enhancer histone marks	DNAse	Proteins bound	Motifs changed	NHGRI/EBI GWAS hits	GRASP QTL hits	Selected eQTL hits	GENCODE genes	dbSNP func annot
2	241835521	0.94	0.97	rs4571087	G	C	0.61	0.63	0.74	0.60			BLD			AP-1			16 hits	8.9kb 5' of AC114730.2	
		0.96	-0.98	rs71052612	CGGTG	C	0.57	0.36	0.29	0.40		9 tissues	13 tissues	BLD,BLD	EGR1	25 altered motifs			1 hit	400bp 5' of AC114730.2	
2	241845788	0.99	1	rs4596012	C	T	0.54	0.62	0.70	0.60			5 tissues			Egr-1,Znf143			14 hits	751bp 3' of AC114730.2	
2	241846001	0.91	0.99	rs7421578	G	A	0.49	0.61	0.69	0.58			5 tissues			4 altered motifs			15 hits	964bp 3' of AC114730.2	
2	241846137	0.99	1	rs7421562	G	T	0.54	0.62	0.70	0.60			5 tissues			4 altered motifs			14 hits	1.1kb 3' of AC114730.2	
2	241849406	1	1	rs7568402	A	G	0.56	0.62	0.72	0.60			4 tissues			6 altered motifs			14 hits	474bp 3' of PDCD1	
2	241851121	1	1	rs2227981	A	G	0.54	0.62	0.72	0.60			ESDR, IPSC, LIV			5 altered motifs			14 hits	PDCD1	synonymous
2	241851407	1	1	rs6705653	T	C	0.53	0.62	0.72	0.60			IPSC			ZBTB7A			15 hits	PDCD1	intronic
2	241854422	0.96	1	rs7419870	G	A	0.54	0.61	0.72	0.59			10 tissues			4 altered motifs			15 hits	PDCD1	intronic
2	241854508	0.98	1	rs6605258	A	G	0.81	0.66	0.72	0.60			10 tissues			8 altered motifs			15 hits	PDCD1	intronic
2	241855866	0.86	0.98	rs6710479	T	C	0.36	0.42	0.24	0.57		BLD	4 tissues			RREB-1,SP1			12 hits	PDCD1	intronic
2	241856465	0.92	0.98	rs6758577	G	T	0.48	0.61	0.73	0.59		BLD	BLD, FAT			7 altered motifs			14 hits	PDCD1	intronic
2	241856491	0.9	0.98	rs6747063	C	G	0.40	0.60	0.73	0.59		BLD	BLD, FAT	SKIN		Mef2,Pou2f2			12 hits	PDCD1	intronic
2	241857486	0.87	0.96	rs13023138	G	C	0.49	0.45	0.24	0.58		4 tissues	BLD, SKIN, SPLN			11 altered motifs			10 hits	PDCD1	intronic
2	241878871	0.81	0.92	rs6605263	C	T	0.62	0.68	0.79	0.61			MUS	MUS,GI		5 altered motifs			9 hits	2.5kb 5' of AC131097.3	
2	241880721	0.83	0.94	rs12998656	T	C	0.62	0.69	0.79	0.61		BLD	12 tissues	BLD		INSM1			11 hits	641bp 5' of AC131097.3	
2	241880865	0.82	0.93	rs28670476	T	C	0.62	0.69	0.79	0.61		13 tissues	15 tissues	BLD		7 altered motifs			12 hits	497bp 5' of AC131097.3	
2	241882321	0.83	0.94	rs6605265	G	A	0.65	0.68	0.79	0.61		21 tissues	12 tissues	20 tissues	5 bound proteins	Mrg1::Hoxa9,Pbx3,TR4			14 hits	AC131097.3	
2	241882326	0.83	0.94	rs6605266	C	G	0.65	0.68	0.79	0.61		21 tissues	12 tissues	21 tissues	4 bound proteins	Mrg1::Hoxa9			14 hits	AC131097.3	
2	241882823	0.83	0.94	rs6605267	A	G	0.68	0.69	0.79	0.61		14 tissues	17 tissues			Ik-1,SRF			16 hits	AC131097.3	
2	241885406	0.83	0.94	rs6605269	T	C	0.69	0.69	0.79	0.62		PANC	10 tissues	PANC		13 altered motifs			16 hits	AC131097.3	
2	241885437	0.83	0.94	rs6605270	T	C	0.69	0.69	0.79	0.62		PANC	10 tissues			NF-E2			16 hits	AC131097.3	
2	241885676	0.83	0.94	rs6732041	T	C	0.69	0.69	0.79	0.62		4 tissues	10 tissues	5 tissues		AhR::Arnt,Arnt,GR			16 hits	AC131097.3	

D

cancers

MDPI

Query SNP: rs10204525 and variants with r^2 >= 0.8

chr	pos (hg38)	LD (r^2)	LD (D')	variant	Ref	Alt	AFR freq	AMR freq	ASN freq	EUR freq	SiPhy cons	Promoter histone marks	Enhancer histone marks	DNAse	Proteins bound	Motifs changed	NHGRI/EBI GWAS hits	GRASP QTL hits	Selected eQTL hits	GENCODE genes	dbSNP func annot
2	241840582	0.81	0.97	rs79245503	C	T	0.17	0.15	0.23	0.11			BLD			5 altered motifs			1 hit	3.8kb 5' of AC114730.2	
2	241846376	0.8	0.97	rs11495865	G	A	0.03	0.07	0.15	0.11			5 tissues			5 altered motifs			3 hits	1.3kb 3' of AC114730.2	
2	241848419	0.8	0.93	rs4973659	G	A	0.16	0.15	0.24	0.11						Pbx3,p300			2 hits	1.5kb 3' of PDCD1	
2	241850169	1	1	rs10204525	C	T	0.39	0.35	0.70	0.12						5 altered motifs			2 hits	PDCD1	3'-UTR
2	241851697	0.82	0.99	rs41386349	G	A	0.17	0.15	0.23	0.11			ESDR, IPSC			4 altered motifs			3 hits	PDCD1	intronic
2	241853775	0.86	1	rs79620053	C	T	0.14	0.14	0.23	0.11			7 tissues			MIF-1,Zfx			3 hits	PDCD1	intronic
2	241866859	0.83	0.94	rs80154873	T	C	0.25	0.29	0.64	0.12		THYM	9 tissues	6 tissues		PU.1,STAT			2 hits	2.7kb 5' of CXXC11	
2	241867263	0.86	0.94	rs4072221	A	G	0.43	0.30	0.64	0.12		BLD, THYM	7 tissues	THYM		GATA,Hoxa7,TAL1			1 hit	2.3kb 5' of CXXC11	
2	241868028	0.83	0.92	rs78574057	G	A	0.23	0.29	0.62	0.12		BLD, THYM, SPLN	6 tissues	THYM,LIV		CTCF,MOVO-B,Nanog			2 hits	1.6kb 5' of CXXC11	
2	241869928	0.87	0.95	rs4973697	T	C	0.24	0.28	0.63	0.12		SKIN, BRN	6 tissues			7 altered motifs			2 hits	CXXC11	intronic
2	241871050	0.84	0.95	rs28512331	G	A	0.11	0.27	0.63	0.12		THYM	9 tissues	4 tissues		BCL,NRSF			4 hits	CXXC11	intronic
2	241871053	0.83	0.94	rs28380696	A	G	0.41	0.30	0.63	0.12		THYM	9 tissues	4 tissues		Sin3Ak-20			2 hits	CXXC11	intronic
2	241871147	0.83	0.91	rs28682292	T	C	0.44	0.33	0.68	0.12		THYM	9 tissues	6 tissues		ERalpha-a,Pax-2,SEF-1			2 hits	CXXC11	intronic
2	241872131	0.88	0.96	rs28499542	C	T	0.23	0.28	0.63	0.12			7 tissues			4 altered motifs			3 hits	CXXC11	synonymous
2	241872208	0.88	0.96	rs28378061	T	C	0.23	0.28	0.63	0.12			7 tissues			7 altered motifs			3 hits	CXXC11	missense
2	241872311	0.87	0.95	rs28523279	A	T	0.34	0.29	0.63	0.12			6 tissues	BLD					2 hits	CXXC11	synonymous
2	241874009	0.88	0.96	rs28620415	G	A	0.23	0.28	0.63	0.12			BLD, SKIN			Pax-6,ZID			3 hits	185bp 3' of CXXC11	
2	241876432	0.87	0.96	rs28663478	T	C	0.34	0.29	0.61	0.12			IPSC, BLD, MUS	BLD,BLD,OVRY	CTCF	PLAG1			3 hits	2.6kb 3' of CXXC11	
2	241876445	0.87	0.96	rs28441154	A	G	0.34	0.29	0.62	0.12			IPSC, BLD, MUS	BLD,BLD,OVRY					3 hits	2.6kb 3' of CXXC11	

E

Query SNP: rs7421861 and variants with r^2 >= 0.8

chr	pos (hg38)	LD (r^2)	LD (D')	variant	Ref	Alt	AFR freq	AMR freq	ASN freq	EUR freq	SiPhy cons	Promoter histone marks	Enhancer histone marks	DNAse	Proteins bound	Motifs changed	NHGRI/EBI GWAS hits	GRASP QTL hits	Selected eQTL hits	GENCODE genes	dbSNP func annot
2	241841903	0.95	0.98	rs78707837	G	A	0.08	0.24	0.13	0.35			4 tissues	MUS		HNF4,Spz1			6 hits	2.5kb 5' of AC114730.2	
2	241845175	0.98	-0.99	rs62193128	C	T	0.92	0.76	0.87	0.65		5 tissues	16 tissues	KID		Smad3,Smad			4 hits	138bp 3' of AC114730.2	
2	241849586	0.86	1	rs28570544	C	T	0.20	0.20	0.13	0.31			5 tissues			15 altered motifs			7 hits	294bp 3' of PDCD1	
2	241853160	1	1	rs7419333	C	G	0.21	0.24	0.12	0.34			IPSC, SKIN, MUS			GATA,PU.1,Zbtb3			6 hits	PDCD1	intronic
2	241853198	1	1	rs7421861	A	G	0.21	0.24	0.12	0.34			IPSC, SKIN, MUS			9 altered motifs			8 hits	PDCD1	intronic
2	241853266	0.98	1	rs5839829	GT	G	0.13	0.23	0.12	0.34			4 tissues			4 altered motifs			6 hits	PDCD1	intronic
2	241853521	0.99	1	rs7420347	G	C	0.08	0.23	0.12	0.34			4 tissues			10 altered motifs			7 hits	PDCD1	intronic
2	241854421	0.85	0.96	rs201546955	C	CA	0.21	0.27	0.15	0.32			10 tissues			5 altered motifs			4 hits	PDCD1	intronic
2	241854424	0.98	1	rs41414844	C	G	0.20	0.24	0.12	0.34			10 tissues			4 altered motifs			7 hits	PDCD1	intronic

Figure S1. Linkage disequilibrium (LD) blocks generated with application of HaploReg v4.1 presenting SNPs being in strong LD (r^2>0.8) with A) rs36084323G>A (PD-1.1). B) rs11568821G>A (PD-1.3). C) rs2227981C>T (PD-1.5). D) rs10204525G>A (PD-1.6). E) rs7421861T>C.

cancers

MDPI

Query SNP: rs822335 and variants with r^2 >= 0.8

chr	pos (hg38)	LD (r^2)	LD (D')	variant	Ref	Alt	AFR freq	AMR freq	ASN freq	EUR freq	SiPhy cons	Promoter histone marks	Enhancer histone marks	DNAse	Proteins bound	Motifs changed	NHGRI/EBI GWAS hits	GRASP QTL hits	Selected eQTL hits	GENCODE genes	dbSNP func annot
9	5448218	1	1	rs822335	T	C	0.62	0.56	0.36	0.63		BLD	7 tissues	8 tissues		6 altered motifs			2 hits	2.3kb 5' of CD274	
9	5449154	1	1	rs822337	T	A	0.49	0.54	0.36	0.63		BLD	7 tissues	BLD,BLD					2 hits	1.3kb 5' of CD274	

Query SNP: rs4143815 and variants with r^2 >= 0.8

chr	pos (hg38)	LD (r^2)	LD (D')	variant	Ref	Alt	AFR freq	AMR freq	ASN freq	EUR freq	SiPhy cons	Promoter histone marks	Enhancer histone marks	DNAse	Proteins bound	Motifs changed	NHGRI/EBI GWAS hits	GRASP QTL hits	Selected eQTL hits	GENCODE genes	dbSNP func annot
9	5468257	1	1	rs4143815	G	C	0.02	0.39	0.57	0.33				BLD		7 altered motifs				CD274	3'-UTR

Query SNP: rs4742098 and variants with r^2 >= 0.8

chr	pos (hg38)	LD (r^2)	LD (D')	variant	Ref	Alt	AFR freq	AMR freq	ASN freq	EUR freq	SiPhy cons	Promoter histone marks	Enhancer histone marks	DNAse	Proteins bound	Motifs changed	NHGRI/EBI GWAS hits	GRASP QTL hits	Selected eQTL hits	GENCODE genes	dbSNP func annot
9	5470497	1	1	rs4742098	A	G	0.13	0.36	0.53	0.29						Pbx3				CD274	3'-UTR
9	5470813	0.89	1	rs4742099	G	A	0.04	0.34	0.53	0.27			BLD, MUS			EWSR1-FLI1				246bp 3' of CD274	
9	5470954	0.89	1	rs4742100	A	C	0.19	0.35	0.53	0.27			BLD, MUS	BLD,SKIN						387bp 3' of CD274	
9	5471271	0.89	1	rs58817806	A	G	0.04	0.34	0.53	0.27			BLD			6 altered motifs				704bp 3' of CD274	
9	5472379	0.84	1	rs10815228	T	C	0.06	0.35	0.51	0.26						Pax-5			1 hit	1.8kb 3' of CD274	
9	5473050	0.88	1	rs148602745	C	T	0.03	0.34	0.53	0.27						7 altered motifs				2.5kb 3' of CD274	
9	5474598	0.89	1	rs140533307	G	A	0.15	0.35	0.53	0.27						15 altered motifs				4kb 3' of CD274	
9	5475754	0.89	1	rs10121574	G	A	0.14	0.35	0.53	0.27				BLD,BRN		20 altered motifs				5.2kb 3' of CD274	
9	5477211	0.88	1	rs10975129	C	A	0.03	0.34	0.53	0.27						4 altered motifs				6.6kb 3' of CD274	
9	5477324	0.88	1	rs62560216	C	T	0.03	0.34	0.52	0.27						Pax-6				6.8kb 3' of CD274	
9	5477766	0.88	0.99	rs12685848	G	A	0.04	0.34	0.53	0.27						5 altered motifs				7.2kb 3' of CD274	
9	5478044	0.89	1	rs4742102	A	G	0.04	0.34	0.53	0.27			BLD			7 altered motifs				7.5kb 3' of CD274	
9	5479640	0.89	1	rs10815230	T	G	0.22	0.36	0.52	0.27						GATA				9.1kb 3' of CD274	
9	5479822	0.89	1	rs7043593	A	G	0.22	0.37	0.53	0.27			BLD							9.3kb 3' of CD274	
9	5480872	0.89	1	rs10815232	A	T	0.15	0.36	0.52	0.27				BLD		CEBPB,Cdc5			1 hit	10kb 3' of CD274	

Query SNP: rs10815225 and variants with r^2 >= 0.8

chr	pos (hg38)	LD (r^2)	LD (D')	variant	Ref	Alt	AFR freq	AMR freq	ASN freq	EUR freq	SiPhy cons	Promoter histone marks	Enhancer histone marks	DNAse	Proteins bound	Motifs changed	NHGRI/EBI GWAS hits	GRASP QTL hits	Selected eQTL hits	GENCODE genes	dbSNP func annot
9	5446078	0.89	0.95	rs10975121	A	G	0.22	0.07	0.08	0.12				7 tissues	PU1	10 altered motifs		1 hit		4.4kb 5' of CD274	
9	5450497	1	1	rs10815225	G	C	0.25	0.08	0.08	0.12				52 tissues	32 bound proteins	12 altered motifs				5bp 5' of CD274	

Figure S2. Linkage disequilibrium (LD) blocks generated with application of HaploReg v4.1 presenting SNPs being in strong LD (r^2>0.8) with rs822335C>T. rs4143815G>C. rs4742098A>G. rs10815225G>C.

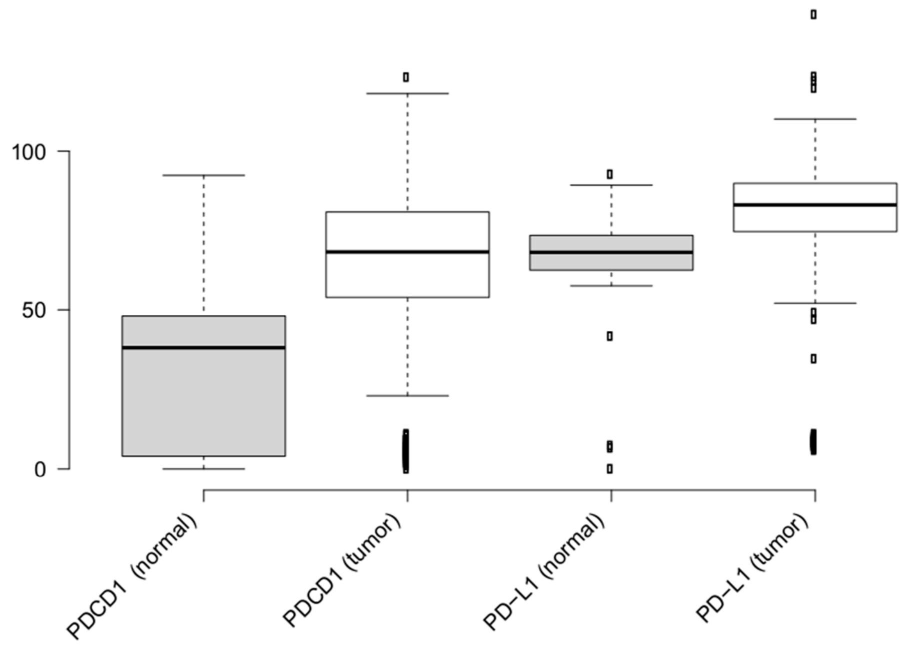

Figure S3. *PDCD1* and *PD-L1* overexpression in ccRCC tissues as compared to control tissues.

© 2020 by the authors. Submitted for possible open access publication under the terms and conditions of the Creative Commons Attribution (CC BY) license (http://creativecommons.org/licenses/by/4.0/).
